# Multidisciplinary approach to improve spontaneous ADR reporting in the pediatric outpatient setting: a single-institute experience in Korea

**DOI:** 10.1186/s40064-016-3151-z

**Published:** 2016-08-30

**Authors:** Hyun Jeong Baek, Yoon Sook Cho, Kwi Suk Kim, Jin Lee, Hye Ryun Kang, Dong In Suh

**Affiliations:** 1 Department of Pharmacy, Seoul National University Hospital, 101 Daehak-ro, Jongno-gu, Seoul, 03080 Republic of Korea; 2 Legional Drug Safety Monitoring Center, Seoul National University Hospital, 101 Daehak-ro, Jongno-gu, Seoul, 03080 Republic of Korea; 3 Division of Allergy, Department of Internal Medicine, Seoul National University Hospital, 101 Daehak-ro, Jongno-gu, Seoul, 03080 Republic of Korea; 4 Department of Pediatrics, Seoul National University Hospital, 101 Daehak-ro, Jongno-gu, Seoul, 03080 Republic of Korea

**Keywords:** Pharmacovigilance, Adverse drug reaction, Child, Outpatients, Korea

## Abstract

In order to improve the reporting of adverse drug reactions (ADRs) as part of the routine practice at the pediatric outpatient department (OPD), we modified our ADR reporting strategy into one that facilitates the reporting process by means of a multi-disciplinary approach. In this study, we retrospectively reviewed ADR records during the period from March to September 2014 when we changed our reporting process as a part of institutional quality assurance (QA) activity. Yearly differences in the number and composition of ADRs were compared, and the descriptive analyses were done for cases reported from OPD during the QA activity in terms of the suspected drugs, type, causality, and severity of ADRs. There were 1211 pediatric ADR reports including 520 cases with underlying hemato-oncologic diseases during the period of 2014. Among the 691 non-oncologic cases, 76 were reported from the OPD, which was a significant increase (347 %) from the 17 cases reported during the previous year. Further analyses of these 76 cases revealed that the caregivers (47.4 %) initiated about half of the reports, the most frequently affected organ was the skin (32.9 %), and the most frequent suspected drugs were anticonvulsants (14.5 %). In contrast to the in-ward system, moderate cases were more frequent (51.3 %) than mild ones. In conclusion, this study provides a profile of pediatric ADRs in the OPD, which were largely under-reported during the usual clinical practice. A multi-disciplinary approach would improve spontaneous ADR reporting at the pediatric OPD.

## Background

As children grow and develop, their pharmacodynamic and pharmacokinetic characteristics continuously change until they reach those of adults for whom treatments are often designed (Batchelor and Marriott 2015). Consequently, it is more frequent to find off-label prescriptions in this subset of the population (Kimland and Odlind 2012), who also have fewer tools to ensure homeostasis and are more prone to suffer adverse drug reactions (ADRs). The ADR profile is also different to adults in terms of type and frequency, and more careful monitoring is required (Blake et al. 2014; Yu et al. 2015). In the clinical practice, however, there is a lack of data describing pediatric ADRs, not to mention high quality evidence regarding their underlying mechanisms.

Pharmacovigilance on subjects attending the outpatient department (OPD) is more challenging than on in-patients due to the difficulty for collecting and analysing ADR data. Under a surveillance system based on spontaneous reporting, ADR collection is largely affected by the disease type and the resources available to the physician during the patient visit, i.e. time allowed for each patient, available diagnostic tools, access to an electronic system for recording and reporting ADRs, and a cultural acceptance to discuss the adverse reactions (Vallano et al. 2005; Black et al. 2015). In Korea, medical expenses are largely shared by the governmental insurance system so that patients can easily access tertiary hospitals (Song 2009), which are located mostly in large cities. In turn, such institutions have transformed their practice pattern to maximize the number of treated patients by constraining the required time per subject. As a result, whereas the institutional physicians can efficiently treat patients for a wide variety of illnesses in this short period, they struggle to properly collect and evaluate ADR data associated with their prescriptions. On the other hand, there are further obstacles to assessing ADRs in OPD patients than in in-patients, e.g., they do not remain at the hospital for long, and it is not possible to perform any further laboratory tests or imaging studies (Hazell and Shakir 2006). Finally, reporting ADRs in the OPD is less motivating for physicians since it is not their primary duty and requires an additional time and effort from which they do not obtain immediate feedback. Even for some physicians, reporting is deferred for fear of legal issues (Al-arifi et al. 2015).

There have been a number of reports investigating ADR profiles in the OPD (Digra et al. 2015; Horen et al. 2002; Bourgeois et al. 2009, 2010), but not many are available concerning pediatric populations. In 2014, as part of a program to try to improve quality and safety, our institution ran a campaign to ameliorate ADR reporting in the OPD by replacing the existing surveillance system with a multi-disciplinary one.

In this report, we aim to (1) share the experience of our activities to promote ADR reporting in OPD, (2) compare the numbers of ADR cases before and after modifying the surveillance system, and (3) describe the current profile of ADRs in a Korean tertiary children’s hospital, excluding anti-neoplastic treatments and vaccinations.

## Results

**Fig. 1:**
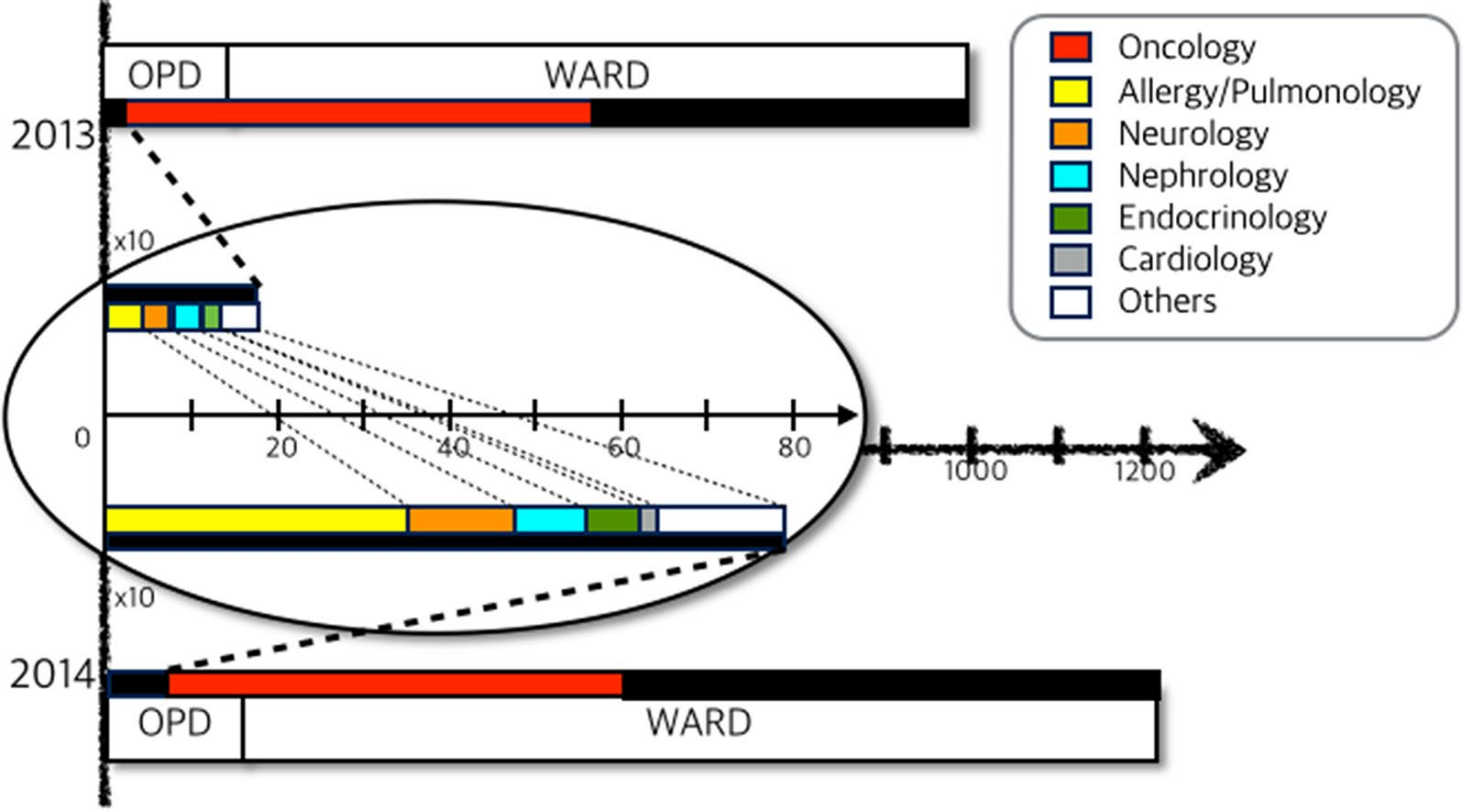
The number of adverse drug reaction (ADR) cases between the two periods. The central part inside the oval is the magnitude of the comparison of ADR numbers according to division of pediatrics except for the oncology one. The *black color* refers to the hospital divisions other than pediatric oncology, in *red*. *Each color* of other specific divisions is listed in the figure

The number of reported ADRs is depicted in Fig. 1. From March 2014 to September 2014 the total number of reported ADRs, including ward and OPD ADRs was 1211, which meant a 21.5 % increase from the 997 reported during the same period in 2013. The number of cases reported in the pediatric oncology division including both the ward and the OPD was 520, which was a 4.1 % decrease from the 542 reported during the same period in the previous year. On the other hand, the number of reported ADRs from divisions other than pediatric oncology was 691, which meant a 51.9 % increase from the 455 reported during the same period in 2013. Our study focused on the 76 cases that were reported from the non-oncological OPD, which meant a 347 % increase in ADR reports compared to the 17 cases reported from March to September in 2013. After classifying the reported cases according to hospital division, we observed that the most cases were reported from the division of allergy/pulmonology, followed by neurology, nephrology, endocrinology, and cardiology. On the other hand, the divisions of gastroenterology and critical medicine did not report any case (Fig. 1).

### Reporting route

When we analysed the ADR cases according to the reporting route, we noticed that various personnel from the routine clinical practice had begun to report ADRs. Previously it was mainly the pharmacist, who reported ADR cases in OPD during their medical chart reviews (Table 1). Seventeen (19.7 %) of 76 ADRs in 2014 were through the claim calls to nurses at OPD, 14 (18.4 %) were claim calls or visits to a pharmacy, and 9 (11.8 %) were through questionnaires, which might have been missed in the previous system and consisted as many as those from the medical personnel (37 and 1 cases from doctors and pharmacist, respectively).

**Table 1:** Number of adverse drug reaction cases classified by subjects who initiated the report

Initiated by	Categories in detail	Case, n	
		2013 March–September	2014 March–September
Medical personnel	Doctors during their routine OPD practice	7	37
	Pharmacists reviewing doctor’s prescriptions	10	
	Pharmacists reviewing a withdrawn prescription		1
Patients/caregivers	Phone calls to nurses at OPD		15
	Phone calls/visits to a pharmacy in hospital		14
Both	Filling up questionnaires at the pharmacy for those who are waiting for their prescription		9
Total		17	76

### ADRs characterization

The main clinical signs and symptoms reported as ADRs are listed in Table 2. Skin problems such as rash or urticaria were the most frequent manifestation, followed by gastrointestinal problems such as nausea, vomiting, diarrhoea, or constipation. Generalized symptoms such as lethargy came in the third position.

**Table 2:** Symptom manifestations of pediatric adverse drug reactions reported at the outpatient department

Manifestation	Cases, n(%)
Rash or urticaria	25 (32.9)
Nausea or vomiting	5 (6.6)
Diarrhea or constipation	5 (6.6)
Lethargy	5 (6.6)
Headache	3 (3.9)
Cough	3 (3.9)
Palpitation	3 (3.9)
Others	27 (35.5)
Total	76 (100.0)

Suspected drugs associated with ADRs are listed in Table 3. Drugs with effects on the nervous system, including anticonvulsants (anatomical therapeutic chemical [ATC] group N), were the most common suspected drugs followed by cardiovascular agents (ATC group C) and hormonal remedies, including systemic steroids (ATC group H). Respiratory drugs, including antihistamines (ATC group R), were the third most common suspected agents followed by antibiotics (ATC group J).

**Table 3:** Types of drugs associated with adverse drug reactions

Drug categories	Anatomical therapeutic chemical group	Case, n (%)
Anticonvulsant	N	11 (14.5)
Cardiovascular	C	10 (13.2)
Steroid/hormonal	H	10 (13.2)
Respiratory/antihistamine	R	8 (10.5)
Antibiotics	J	7 (9.2)
Immunosuppressive	L	5 (6.6)
Others	n/a	25 (32.9)
Total		76 (100.0)

*n/a* Not associated

### Causality and severity

When we classified the causality considering both ADRs and suspected drugs, 55 out of 76 (72.4 %) were categorized as ‘possible’, followed by 18 ‘probable’ cases (23.7 %), and two (2.6 %) ‘definite’ cases. Just one (1.3 %) case was categorized as ‘doubtful’.

When we classified the ADR cases according to the severity, 536 out of 698 (76.8 %) cases were mild, 136 (19.5 %) were moderate and 26 (3.7 %) were severe (Table 4). This was not significantly different from the profile of cases in 2013, 374 out of 470 (79.6 %) cases were mild, 77 (16.4 %) were moderate and 19 (4.0 %) were severe (Chi square test, *P* = 0.347). When we further divided the 2014 cases between ward and OPD cases, mild cases ranked the most frequent in the ward (502/622, 80.7 %) followed by moderate and severe ones. Among the OPD cases, moderate cases were the most common (39/76 cases, 51.3 %), followed by mild and severe ones. The difference in order and composition of severity between the ward and the OPD was statistically significant (Chi square test, *P* < 0.001). When we further review the seriousness in the OPD, 4 and 7 cases experienced a brief life-threatening event or hospitalization. However, no case became dead or permanently disabled.

**Table 4:** Distribution of adverse drug reaction severities by the year and the clinical setting

Severity	2013	2014		
	Total, N = 470	Total, N = 698	Ward, n = 622	Outpatient, n = 76
Mild	374 (79.6)	536 (76.8)	502 (80.7)	34 (44.7)
Moderate	77 (16.4)	136 (19.5)	97 (15.6)	39 (51.3)
Severe	19 (4.0)	26 (3.7)	23 (3.7)	3 (3.9)

All values are number of frequencies (%)

### Off-label prescriptions

When we analysed in detail the 76 ADR cases reported at the OPD according to the prescription label, 54 cases (71 %) were prescribed within label whereas 16 cases (21 %) were off-label prescriptions. The remaining 6 cases (8 %) were not assessed due to lack of detailed information. Among the 16 off-label cases, 4 cases were prescribed out of the age (i.e. zolendronic acid intravenously to a 7 year-old child), 4 cases were out of the dosage or route (i.e. somatropin twice as much dose as recommended), and 3 cases were out of the indication (i.e. losartan for hematura). Interestingly, the other 5 cases were prescribed at a dose lower than the one recommended in the label (Table 5).

**Table 5:** Number of cases according to the type of prescription

	Case, n		Case, %
On label	54		71.1
Off-label	16		21.1
Indication		3	
Age		4	
Dosage/route		4	
Low dose		5	
Data incomplete	6		7.9
Total	76		100.0

## Discussion

The major change in the ADR reporting strategy during the quality assurance (QA) activity was the replacement of the physician by the pharmacist as the main orchestrator of the ADR reporting process. As a result, spontaneous ADR reporting was encouraged and there was an increase in the number of reported ADRs from pediatric patients at the OPD, compared to the previous year. The ADR cases reported at the OPD were different to those reported in the ward regarding severity and suspected drugs. Moreover, the clinical profile of reported ADRs at the OPD was unique from that of other countries, which reflects the characteristic patterns of clinical practice in the Korean children population. This report is the first that described ADR profiles of Korean children treated at the OPD.

The systems in place for collecting ADR data differ between nations, and is inevitably influenced by the pattern of clinical practice and the culture of the country, especially where spontaneous reporting is concerned (Vallano et al. 2005; Margraff and Bertram 2014). In a Korean tertiary children’s hospital, under a medical practice pattern that emphasizes efficiency, it is difficult to gather sufficient information and to properly report ADRs. The time pressure routine does not allow for screening questions, and ADRs go unnoticed unless patients actively voice their complaints. Such a limitation has been acknowledged in previously published qualitative research (Vallano et al. 2005). Moreover, reporting ADRs to the database requires additional efforts with no immediate reward for physicians, which may explain the low ADR reporting frequency recorded during the routine process in 2013.

As part of our study, we tried to improve the ADR reporting in the routine practice by minimizing the reporting process. We used a template for physicians, which required only the minimum essential pieces of data they could gather during the scheduled visit, and we asked the nurses to check the list of suspected cases and transfer them to the pharmacist. Finally, we let the pharmacists review the medical records of the affected subjects and fill up the reporting forms for submission to the database. Once we established the reporting process, claiming calls from the caregivers or patients themselves at the drug-distribution site or at the OPD out of the practice opening hours were also reviewed by the pharmacist. As a result, the total number of ADR reports initiated by the patients or caregivers was almost as high as the ones initiated by the physicians.

Nurses in general wards play an important role as observers and reporters of ADRs. In the OPD their role is different, but it is still important, for they serve as connection between patients, physicians, and pharmacists. As a first point of contact with the outpatient they hold a unique position to be informed of ADRs by the patients themselves. We estimate that about one-fifth of ADRs might have been missed if it had not been for the intervention of the nurses.

Regarding ADR severity in the OPD, moderate cases were more frequent than mild ones, which is different from the in-ward reports. In subjects who admitted to hospital, the medical personnel may easily notice mild ADRs during the observation period. However, mild ADRs may not be noticed in the OPD setting unless they are mentioned by the patients or caregivers. On the other hand, mild ADRs occur mostly briefly so they may not be present at the time of the visit. Considering that moderate ADRs require some degree of medical intervention (Hartwig et al. 1992; Davies et al. 2006), an improved reporting of moderate ADRs may be important to recognize potentially harmful ADRs and the drugs that cause them.

Regarding anatomical location, the skin and gastrointestinal tract were most commonly affected according to previous reports carried out in pediatric patients both in the ward and in OPD settings (Bourgeois et al. 2009; Darnis et al. 2015; Thiesen et al. 2013). One interesting finding of this report is that lethargy, which is otherwise overlooked during the routine practice, was also frequently reported. It implies that in our daily practice we need to ask patients actively about their subjective symptoms as well as their objective ones. In this study, ATC class N (anticonvulsants), R (antihistamines) and J (antibiotics) drugs were frequently reported as the suspected drugs, in agreement with previous reports (Digra et al. 2015; Bourgeois et al. 2009; Jose and Rao 2006; Kaushal et al. 2007). However, vaccines and antipsychotic drugs which had been frequently reported in general (Aagaard and Hansen 2011; Li et al. 2014) were missed out in this study because OPDs in the psychiatric department and routine vaccinations were not included in this QA activity.

We excluded cases reported at the division of pediatric oncology because chemotherapeutics for cancer are different from other drugs in that their recommended doses are determined by maximal tolerable doses and their safety margins are narrow (Mathijssen et al. 2014). This could have blunted the clinical profiles of other medications. On the other hand, pediatric cancer patients are frequently admitted to the in-ward or the day-care ward, where they have a specialized pharmacist who monitors and adjusts the chemotherapeutic regimens. To focus on the changes in the non-oncological OPDs, we did not apply the QA program to the OPD of pediatric oncology.

This study deals with the activity during a 7-month period, which cannot be generalized to the whole year. Especially the seasonal variance of prescriptions and the external effect of social issues related to drug safety could not be reflected on this report. However, the QA program was only supported by the institution for 7 months, and once it finished, we were unable to ensure the participation of all the necessary medical personnel. Therefore, we compared our results to the data available from the previous year during the same period, from March to September 2013.

It was not possible to calculate accurately the amount of actual ADRs occurred during the OPD practice. Although we found a significant increase in the number of ADR reports in 2014 compared to the scanty number in 2013, we assume that many ADRs went unnoticed by the authors. In the pediatric OPD, where a continuous monitoring is impossible and the complaints from kids are nonspecific or vague, a cultural change to report freely ADRs is required. Moreover, we often notice physicians misjudge ADRs as malpractice and actively discard the reporting, this needs to be overcome in the near future in order to develop a successful ADR surveillance system (Vallano et al. 2005).

When we further investigated the prescriptions associated with ADRs, more than two-thirds were within label. Since we lack information on how many prescriptions not associated with ADRs are made off-label, we could not confirm or dismiss the well-known hypothesis that ADRs in children are greater for drugs prescribed off-label than within label (Wallerstedt et al. 2011). Considering that the incidence of off-label prescriptions is lower than that previously reported (Horen et al. 2002), and that 31 % of the off-line prescriptions were at the “less than recommended doses”, which actually suggests that the ADR might also have happened if we prescribed them on-label; the relationship between the off-label prescriptions and ADRs is less likely in OPD subjects. However due to the small sample size, our suggestion cannot be generalized to the larger population.

## Conclusion

In conclusion, this study suggested a “fine-tune” to enhance the ADR monitoring activities. This change in our reporting strategy seems very tiny but is exceptionally effective in medical environments where the physicians are so time-pressed that any activities irrelevant to the clinical practice are deferred. Moreover, this study provided the profile of the spontaneously reported ADR on OPD pediatric subjects in a Korean tertiary hospital. More ideas and trials to promote spontaneous reporting need to be developed and implemented in the clinical practice. The generated evidence will be used to influence governmental policies to ensure safer practices in drug prescription.

## Methods

### Characteristics of the institution

Seoul National University Children’s Hospital is a tertiary general hospital founded 30 years ago, with capacity for 311 beds, including 60 intensive care unit beds. Sixteen divisions provide outpatient services to 313,000 patients yearly. Every year each division of the hospital is encouraged to present an innovative proposal to improve the quality and safety of the clinical practice, this is known as the QA activity. The change in the ADR reporting strategy was a part of QA activity proposed by the pediatric outpatient division, and took place from March 2014 to September 2014. It involved 11 pediatric divisions excluding the pediatric oncology ones, where the ADRs are already being intensively monitored in the day-care ward. The initiative was supported by 27 professors belonging to the 11 specialized pediatric divisions that handle together about 121,000 patient visits per year.

### The ADR monitoring system before and after the QA program

The ADR monitoring system of the institute relied largely on spontaneous reporting, consisting of medical personnel that reported suspected ADRs via the electronic medical report (EMR) system. Reports were collected and comprehensively analysed by the full-time reviewers at the regional monitoring centres. After determining the suspected medicines, type of adverse reactions, causal relationships and outcome, they released the results and recommendations to the first responder as a feedback report.

**Fig. 2:**
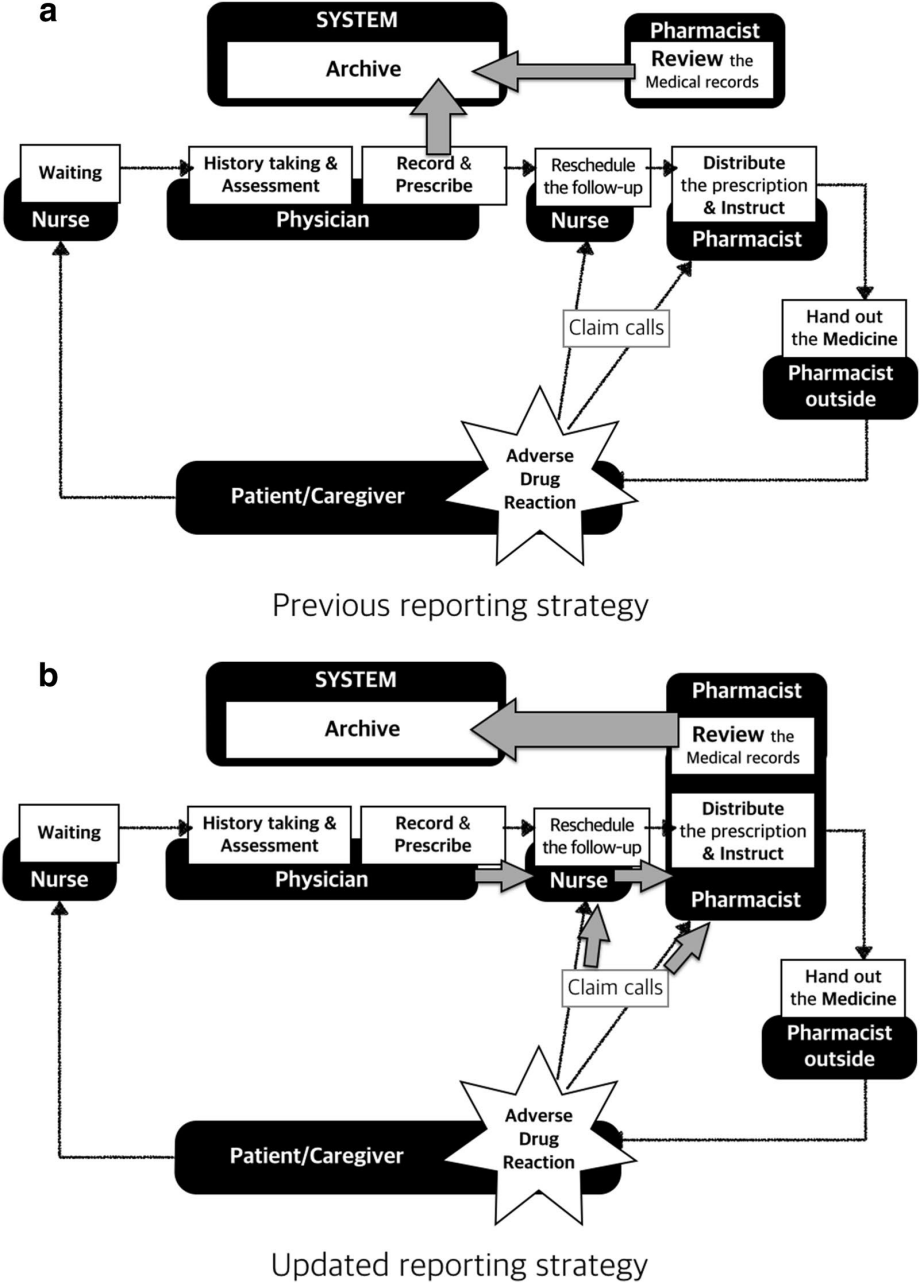
Change in the reporting strategy. In (**a**) the previous strategy, physicians reported ADRs that they came across with during or after their general practice. Claim calls at the outpatient clinic out of service hours or at the drug-distributing site were transferred to the OPD nurse who eventually contacted the physicians and let the claimers get informed about their inquiries. However, during this process no information was sent to the regional ADR monitoring centre. In (**b**) the updated reporting strategy, physician and nurse only had hand over the identifiers to pharmacists, and then they report the relevant cases after reviewing the medical records of the suspected cases

The changes made to the surveillance system during the QA period are depicted in Fig. 2. Originally, the screening and reporting of ADRs in outpatient clinics were done in the same manner as in the in-ward system i.e. physicians would report while carrying out their usual practice, and pharmacists would do it after their retrospective review of prescriptions and EMRs, but this system did not translate into many ADR reports. Moreover, claim calls to the drug-distributing site or the outpatient clinic out of the service hours were transferred to the OPD nurse, who would eventually contact the physicians and let the claimers get informed about their inquiries by them. However, since the physicians were not always available registering the case, this information was not sent to the regional ADR monitoring centre.

In the updated surveillance strategy, nurses and pharmacists played key roles in gathering and reporting ADRs. To minimize any additional burden arising from the reporting process, physicians were asked to provide the relevant information on the medical chart during their usual practice and leave only the list of cases for the attending nurse at the end of their turn. The nurse then transferred it to the pharmacist on duty who would review the medical records of each case and register them into the system. The cases reported through claim calls, whether received at the OPD or the drug-distribution site, were also notified to the pharmacist, who would then record, review, and register the suspected ADR cases. Moreover, as an additional QA feature, we prepared a questionnaire and made it available at the drug-distribution desk for patients to assess their experience with ADRs and allow them to freely communicate suspected cases to us.

Cases archived in the institutional ADR monitoring system were evaluated by specially trained pharmacists in order to identify suspected drugs, type and severity of ADRs, causality, and recommended course of action. The feedback reports were reviewed by the physician in charge of the monitoring system and included in the patient’s medical records.

### Measures

This analysis was a retrospective review of the cases reported during this QA activity. The prescriptions and feedback reports relevant to each case were categorized according to the clinical characteristics of the subjects, the reporting source, the suspected drug, the type and severity of ADRs, and the association with off-label use. Suspected drugs were categorized according to the ATC codes (World Health Organization Collaborating Centre for Drug Statics Methodology 2015). As a first level classification, medicines were divided into 14 main groups.

Causal relationships were classified as ‘definite’, ‘probable’, ‘possible’ and ‘doubtful’ according to the Narajo algorithm (Naranjo et al. 1981). The severity of ADRs was categorized as mild, moderate, and severe based on the classification system proposed by Hartwig et al. (1992) and Davies et al. (2006) and the Common Terminology Criteria for Adverse Events (CTCAE) provided by the National Cancer Institute version 4.03 (National Cancer Institute 2015). Mild ADR referred to cases of Hartwig’s level 1–2, which require no medical intervention or simple discontinuation of suspected drugs. On the other hand, moderate ADR referred to cases of Hartwig’s level 3–4, which require further management such as an administration of antidote or a prolonged hospitalization. Finally, severe ADR referred to more severe cases of Hartwig’s level 5–7, which result in persistent or significant disability that requires intensive medical care, permanent harm, or death (Hartwig et al. 1992; Davies et al. 2006). If the degree of ADR is measurable according to the CTCAE, cases of grade 1–2, 3–5, or 6–7 were considered as the mild, moderate, or severe ADRs.

Off-label prescription was evaluated by verifying prescriptions in terms of the indication, age, dosage, and route. The label was defined as the one recorded by the Korean ministry of Food and Drug Safety database (Korean Ministry of Drug and Food Safety 2015), which is similar to the US food and drug administration (US Food and Drug Administration 2015). Under-dose prescription cases were considered separately since they were not considered of clinical relevance.

While most comparisons were descriptively analysed, the differences in ADR severity between the ward and the OPD in 2013 and 2014 were assessed by Chi square tests. The review board of Seoul National University Hospital which supervises conducts at the Children’s Hospital approved this protocol (IRB Number was 1504-028-662).

## Abbreviations

ADR: adverse drug reaction
ATC: anatomical therapeutic chemical
CTCAE: Common Terminology Criteria for Adverse Events
EMR: electronic medical report
FDA: food and drug administration
OPD: outpatient department
QA: quality assurance
